# Evaluation of the Relationship between Serum Lipid Profile andOral Lichen Planus

**DOI:** 10.15171/joddd.2015.046

**Published:** 2015-12-30

**Authors:** Masoumeh Mehdipour, Ali Taghavi Zenouz, Farnaz Davoodi, Narges Gholizadeh, Hossein Damghani, Sanaz Helli, Maryam Safarnavadeh

**Affiliations:** ^1^Associate Professor, Department of Oral Medicine, Faculty of Dentistry, Shahid Beheshti University of Medical Sciences, Tehran, Iran; ^2^Associate Professor, Department of Oral Medicine, Faculty of Dentistry, Tabriz University of Medical Sciences, Tabriz, Iran; ^3^Postgraduate Student, Department of Operative Dentistry, Faculty of Dentistry, Tabriz University of Medical Sciences, Tabriz, Iran; ^4^Assistant Professor, Department of Oral Medicine, Faculty of Dentistry, Tehran University of Medical Sciences, Tehran, Iran; ^5^Postgraduate Student, Department of Oral Pathology, Faculty of Dentistry, Tabriz University of Medical Sciences, Tabriz, Iran; ^6^Assistant Professor, Department of Oral Medicine, Faculty of Dentistry, Tabriz University of Medical Sciences, Tabriz, Iran; ^7^Assistant Professor, Educational Deputy of Ministry of Health and Medical Education, Tehran, Iran

**Keywords:** Lichen planus, serum, lipid profile

## Abstract

***Background and aims.*** Oral lichen planus (OLP) is an immunologic disorder. A large number of studies have reported that lipid rafts have a key role in receptor signaling of lymphocytes. Here, we explore the potential of lipid rafts as targets for the development of a new class of agents to down-modulate immune responses and treat autoimmune diseases.

***Materials and methods.*** The present cross-sectional study evaluated 88 patients referring to the Department of Oral Medicine in 3 groups (Group 1: erosive OLP; Group 2: non-erosive OLP; Group 3: healthy). A total of 3 mL of blood sample was taken from each subject and the serum levels of cholesterol, triglycerides, HDL and LDL were determined. The mean outcomes of each group were compared with each other and analyzed two by two.

***Results.*** The results of statistical analyses showed no significant differences in mean HDL and LDL serum levels between the three groups. The results of post hoc LSD test showed that mean serum levels of subjects with erosive and non-erosive lichen planus were higher than those in healthy subjects. In relation to triglyceride serum levels, the mean serum levels of triglycerides were higher in erosive and non-erosive OLP patients compared to healthy subjects.

***Conclusion. ***Triglyceride and cholesterol can be considered to have a critical role in the incidence of lichen planus and in its manifestations as predisposing factors.

## Introduction

Oral lichen planus is an immunologically based, chronic, inflammatory mucocutaneous disorder of undetermined etiology. The overall prevalence of lichen planus in the general population is about 0.1-4%. It generally occurs more commonly in females at a ratio of 3:2, and most cases are diagnosed between the ages of 30 and 60, but it can occur at any age. It is a relatively common disorder affecting stratified squamous epithelia. It is of special importance due to its malignant potential and can be a source of morbidity. OLP can develop on any mucosal surface, including the larynx and esophagus, but lesions have a predilection for the posterior buccal mucosa. The specific etiology of oral lichen planus is unknown. It is believed to result from an abnormal cell-mediated immune response with infiltrating cell population composed of both T4 and T8 lymphocytes in the basal epithelial cells. They are recognized as foreign because of changes in the antigenicity of their cell surfaces.^1^

Based on the pathogenesis, biomarkers are being proposed to predict the onset and severity of oral lichen planus, like CD275 and desmogleins 1 and 3, and to detect malignant transformation.^2^

Activated DCs (dendritic cells) may be instrumental in the presentation of autoantigens contributing to the activation of T cells.^3^

Recognition of antigen/MHC complexes by the TCR is assisted by co-receptors, CD4 and CD8, and co-stimulatory receptors such as CD28, expressed on the surface of T cells that pair with their cognate ligands on the APC named the immunological synapse.^4^

The term lipid raft is used to describe microdomains in the plasma membrane that are in liquid-ordered phase owing to their lipid composition which is rich in cholesterol, glycosphingolipids and sphingomyelin.^5^

A large number of studies have reported that lipid rafts have a key role in receptor signaling and activation of lymphocytes. In T cells, lipid raft involvement was demonstrated in the early steps during T cell receptor (TCR) stimulation.^6^

The importance of lipid raft signaling in the pathogenesis of a variety of conditions such as Alzheimer’s, Parkinson’s, cardiovascular and prion diseases, systemic lupus erythematosus and HIV has been elucidated in recent years, making these specific membrane domains an interesting target for pharmacological approaches in the cure and prevention of these diseases.^7^

There are documented defects in the expression and function of various signaling molecules and pathways proximal to the TCR in SLE T cells. Compared to healthy controls, lipid rafts from SLE T cells were found to contain higher amounts of CD45 and co-immunoprecipitation experiments revealed that a larger fraction of this pool was associated with Lck (lymphocyte-specific protein tyrosine kinase). As a consequence more Lck in lipid rafts was in the active form. Therefore, an increase in the localization of CD45 into lipid rafts is a key step for Lck conversion to the active form and may be a critical step in the activation of T cells.^8,9^

In accordance, SLE T cells when stimulated via the TCR (T cell receptors), are poor activators of MAPK (mitogen-activated protein kinase) pathways and hypoproliferate in vitro*.*^10^

A supposition of the lipid raft theory is that cholesterol and certain other lipids play a preliminary role in the formation of membrane domains. It is possible that changes in lipid composition modify membrane organization by increasing the abundance or the size of lipid raft domains. Recently, a case-control study found that lichen planus was associated with dyslipidemia in a large series of patients.^11^

Hyperlipidemia involves abnormally elevated levels of some or all the lipids and/or lipoproteins in the blood. It is the most common form of dyslipidemia. It usually means that you have high cholesterol and high triglyceride levels. There are really two major causes. One is diet from animal products (meat, cheese, milk, etc). Primary (genetic) causes and secondary (lifestyle and other) causes contribute to dyslipidemias in varying degrees. For example, in familial combined hyperlipidemia, expression may occur only in the presence of significant secondary causes. Other diseases such as thyroid problems or diabetes have roles, too. A linear relation exists between lipid levels and cardiovascular risk. Higher levels of the “good" HDL cholesterol are associated with decreased risk of heart disease and stroke, which slows the development of plaque. The “bad” LDL cholesterol, on the other hand, can lead to blockages if there’s too much in the body.^12^

Here, we discuss these findings and explore the potential of lipid rafts as targets for the development of a new class of agents to down-modulate immune responses and treat autoimmune diseases.

In the majority of previous studies dyslipidemia has been evaluated but not separately. In this study we evaluated all the lipid elements; HDL, LDL, triglyceride and cholesterol.

This study was undertaken to answer the following questions:

- Does serum lipid affect the occurrence of oral lichen planus?
- Which type of serum lipid mostly affects the disease?
- Can we consider dyslipidemia as a possible risk factor for OLP?
- The aim of this study was to evaluate the relationship between serum lipid profile and oral lichen planus; therefore, in case of any possible positive relationship, controlling these serum elements would decrease the incidence of oral lichen planus.

## Materials and Methods

The subjects in the present cross-sectional study consisted of 88 patients referring to the Department of Oral Medicine, Faculty of Dentistry, divided into 3 groups (Group 1: erosive OLP; Group 2: non-erosive OLP; Group 3: healthy). Forty-four subjects had oral lichen planus, 22 of whom had erosive and 22 had non-erosive type of oral lichen planus; 44 subjects without any specific disease were selected as the control group (23 females and 21 males, with a mean age of 36.6±11.7 years). The subjects in both the test and control groups were matched in relation to age (an age range of 20-60 years; mean ± standard deviation = 35.7±8.5 years) and sex (46 males, 42 females). The subjects were selected serially.

### Inclusion Criteria 

A definitive diagnosis of oral lichen planus based on clinical/pathological view; an age range of 18-60 years at affliction; and signing an informed consent form to participate in the study.

### Exclusion Criteria

Presence of any factors resulting in lichenoid reactions; congenital and acquired defects of the immune system such as AIDS, chemotherapy, intravenous drug abuse, hemophilia and those undergoing hemodialysis; any contraindication for biopsies of lesions that needed a biopsy procedure for diagnosis; patients with dysplasia being reported in histopathological evaluations of their lesions; unwillingness to participate in the study; use of tobacco or cigarette smoking; use of drugs affecting the serum lipid profile; and drug therapy for lichen planus during the previous two months.

### Study Procedures

A total of 3 mL of blood sample was taken from each subject. The serum levels of cholesterol, triglycerides and high-density lipoproteins (HDL) were determined by a piece of Hitachi test equipment (Model 917, Germany) using a triglyceride kit (Aria Pharma Kit) and HDL kit (Pars Acmous Rouyaben). The serum levels of low-density lipoproteins (LDL) were determined using the kit provided by Pars Azmoun; the kit eliminates all the lipoproteins except for LDL and determines its serum levels by an enzymatic technique.

### Statistical Analysis

Data were analyzed with descriptive statistical methods and Manova test using SPSS 15; since more than one variable were continuous and dependent, the mean differences test was used to compare the differences between two groups of erosive lichen planus, non-erosive lichen planus and one control group. In addition, in cases in which the results of Manova test showed statistically significant differences in the means between the groups a suitable post hoc test (such as LSD) was used to determine which two groups exhibited significant differences. Statistical significance was defined at P<0.05 (Table 1).

**Table 1:** The results of Manova test

**Variables**	**Degree of freedom**	**F**	**P**
**HDL**	2	1.635	0.201
**LDL**	2	0.369	0.692
**Cholesterol**	2	4.48	0.014
**Triglyceride**	2	10.436	0.00

### Ethical Considerations 

Both the test and control subjects participated in the study voluntarily by signing an informed consent form.

## Results

All of our case and control group patients continued and finished the steps of the study.

### Descriptive Data of the Variables

The mean serum cholesterol levels in subjects with erosive and non-erosive lichen planus and in healthy subjects were 200, 198 and 172.3 mg/dL, respectively. The mean HDL serum levels in subjects with erosive and non-erosive lichen planus and in healthy subjects were 48.5, 46.9 and 53.6 mg/dL, respectively. The mean LDL serum levels in subjects with erosive and non-erosive planus and in healthy subjects were 97.5, 97.9 and 102 mg/dL, respectively. The mean triglyceride serum levels in subjects with erosive and non-erosive lichen planus and in healthy subjects were 128.5, 144.3 and 86.2 mg/dL, respectively.

The results of statistical analyses showed no significant differences in mean HDL and LDL serum levels between the three groups evaluated (P_cholesterol_ = 0.014; P_triglyceride_ = 0.00). Post hoc LSD test was used because at a significance level of P<0.05 it is the best choice for a post hoc test. The results of this test are presented in Table 2.

**Table 2:** The results of post hoc LSD test for the evaluation of cholesterol and triglyceride serum levels

**Variable**	**Group pairs**		**Means differences**	P^*^
**Cholesterol**	1	2	2.3	0.857
		3	28.5	0.013
	2	3	25.9	0.022
**Triglyceride**	1	2	5.5	0.77
		3	63.6	0.00
	2	3	58	0.001
^*^Group 1: erosive; Group 2: non-erosive; Group 3: healthy				

**Figure 1.**
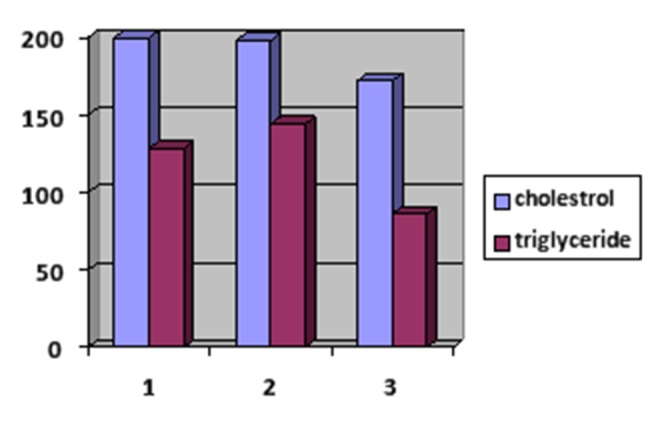
The profile plate of cholesterol and triglyceride levels in the study groups (1: erosive lichen planus; 2: non-erosive lichen planus; 3: control).

The results of LSD test showed significant differences in cholesterol serum levels between groups 1 and 3 and groups 2 and 3; in other words, mean serum levels of subjects with erosive and non-erosive lichen planus were higher than those in healthy subjects. In relation to triglyceride serum levels, there were significant differences between groups 1 and 3 and groups 2 and 3; in other words, the mean serum levels of triglycerides were higher in erosive and non-erosive lichen planus patients compared to healthy subjects as shown in Figure 1.

**Figure 2.**
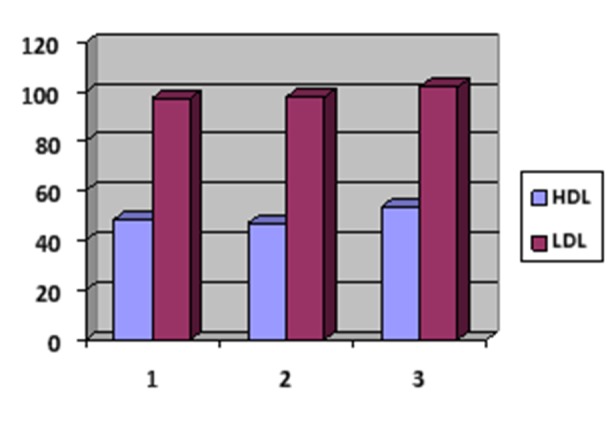
The profile plate of LDL and HDL levels in the study groups (1: erosive lichen planus; 2: non-erosive lichen planus; 3: control).

The results of statistical analysis showed no significant differences in mean HDL and LDL serum levels between the three groups. Figure 2presents serum LDL and HDL profiles in the study groups.

## Discussion

The results of the present study are consistent with those of a study by Arias et al^14^ in Spain. They reported a higher than normal levels of triglyceride and total cholesterol in patients with lichen planus but they reported HDL levels less than the normal levels. They observed exclusion criteria such as the use of medications. It should be remembered that an increase in HDL levels can have an inhibitory effect on cytokines such as interferon-α which is an inflammatory mediator involved in the initiation of clinical symptoms and signs of lichen planus. A decrease in HDL levels might in itself be a sign of this trend, and in the present study higher than normal levels were not reported.

In addition, a study by Gupta et al^15^ in India, in which the serum lipid profiles in precancerous lesions such as leukoplakia lichen planus were evaluated, showed a decrease in cholesterol and triglyceride levels in the case group compared to the control group, contrary to the results of the present study. Gupta et al^15^ attributed this decrease in lipid profile to a loss of cell integrity. The results of that study indicated that lipids have a strong relationship with the health status of subjects. They concluded that cell division rate in malignant conditions decreases lipid profile in the tissue undergoing proliferation. Compared to the results of the present study it might be concluded that there were no proliferations and dysplasia in the subjects evaluated in the present study, which is contrary to the results of studies by Lohe et al,^16^ and Chalkoo et al,^17^ who reported that the total cholesterol levels of the serum had an inverse relationship with the incidence of precancerous lesions such as leukoplakia and lichen planus.

In similar diseases with histopathological characteristics similar to those of lichen planus, such as psoriasis, too, such evaluations have been carried out and contradictory results have been reported.

The homeostasis of the immune system is stringently controlled by the specificity and fidelity of lymphocyte activation. In autoimmune diseases this specificity and fidelity is compromised, leading to pathology. Whether changes in the composition or structure of lipid rafts play a role in autoimmunity is an important question which has started to be addressed in the last few years. It was noted that human T cells activated in vitro via their TCR synthesize more GM1 lipid, a component of raft domains, as detected by staining with CTB.^12^

Recent studies have demonstrated the incorporation of dietary PUFAs into membranes of T and B lymphocytes, and, subsequently, alterations in the lipid raft phospholipids of these cell types. These changes in membrane lipid composition have further been shown to affect localization of immunogenic receptors, such as IL-2 (interleukin 2) and FcR (Fc receptor), to lipid rafts and altered activation of their downstream mediators, such as PKC*θ* (protein kinase C*θ*) and the transcription factors NF-*κ*B (nuclear factor *κ*B) and AP-1 (activator protein 1). These findings imply a link between a PUFA-mediated immune response and altered downstream signaling of raft-associated signaling molecules.^6^

In the majority of previous studies,^13-18^ either serum lipid levels have been evaluated thoroughly, such as a study by Dreiher &amp; Cohen,^13^ or several patients have been evaluated simultaneously, such as a study by Yadav et al.^18^ Therefore, in order to minimize these limitations, in the present study, the lipid profiles were evaluated thoroughly in patients with lichen planus. The results of the present study showed that in patients with erosive lichen planus the lipid profile, including triglyceride and total cholesterol levels, were high, with no significant differences between patients with erosive and non-erosive lichen planus, consistent with the results of a study by Dreiher et al,^11^ in which only the serum levels of cholesterol and triglyceride were evaluated and significant differences were observed between patients with lichen planus and the healthy controls. Dreiher &amp; Cohen^13^ did not evaluate other criteria of serum lipids; in addition, the lichen planus type was not evaluated. However, it was pointed out that with an increase in the severity of lesions some serum levels of lipids decrease, such as total cholesterol, triglyceride and HDL as favorable cholesterol.^13^

According to the results of this study, dyslipidemia affects the occurrence of OLP and cholesterol and triglyceride are the most important elements in this aspect; therefore, it can be considered a risk factor for erosive OLP.

It is suggested that in future studies on patients with OLP, who have abnormal lipid profile, the lipid profiles be restored to normal and its effect on recovery from lichen planus be evaluated.

## Conclusion

Based on the results of the present study triglyceride and cholesterol can be considered to have a critical role in the incidence of lichen planus and in its manifestations as predisposing factors.

## Acknowledgments

The authors would like to thank Tabriz University of Medical Sciences and Research Vice Dean of Faculty of Dentistry for their assistance in carrying out the experiments. The authors declare that they have no competing interests.
